# Prognosis value of microscopic bile duct invasion in hepatocellular carcinoma: A multicenter study

**DOI:** 10.1002/cam4.6650

**Published:** 2023-11-01

**Authors:** Qizhen Huang, Kongying Lin, Zhipeng Lin, Hongbin Ji, Xiaoyu Zhou, Bin Wang, Yufeng Chen, Chuandong Sun, Shuguo Zheng, Jinhong Chen, Yifan Wang, Yanming Zhou, Weiping Zhou, Yongyi Zeng

**Affiliations:** ^1^ Department of Radiation Oncology Mengchao Hepatobiliary Hospital of Fujian Medical University Fuzhou China; ^2^ Department of Hepatopancreatobiliary Surgery Mengchao Hepatobiliary Hospital of Fujian Medical University Fuzhou China; ^3^ The School of Basic Medical Sciences Fujian Medical University Fuzhou China; ^4^ Department of Pathology Mengchao Hepatobiliary Hospital of Fujian Medical University Fuzhou China; ^5^ Department of Hepatopancreatobiliary Surgery Zhangzhou Affiliated Hospital of Fujian Medical University Zhangzhou China; ^6^ Department of Hepatobiliary and Pancreatic Surgery Affiliated Hospital of Qingdao University Qingdao China; ^7^ Institute of Hepatobiliary Surgery, Southwest Hospital Third Military Medical University Chongqing China; ^8^ Department of General Surgery Huashan Hospital, Fudan University Shanghai China; ^9^ Department of General Surgery, Sir Run Run Shaw Hospital, School of Medicine Zhejiang University Hangzhou China; ^10^ Department of Hepato‐Biliary‐Pancreato‐Vascular Surgery First Affiliated Hospital of Xiamen University Xiamen China; ^11^ The Third Department of Hepatic Surgery, Eastern Hepatobiliary Surgery Hospital Second Military Medical University Shanghai China; ^12^ Department of Hepatopancreatobiliary Surgery First Affiliated Hospital of Fujian Medical University Fuzhou China

**Keywords:** bile duct invasion, hepatocellular carcinoma, survival, vascular invasion

## Abstract

**Objective:**

To evaluate the prognostic significance of microscopic bile duct invasion (MiBDI) in hepatocellular carcinoma (HCC) following R0 resection.

**Patients and Methods:**

Patients who underwent R0 resection for HCC at nine medical centers were stratified into five groups: neither bile duct nor vascular invasion (MiBDI−MVI−), microscopic bile duct invasion alone (MiBDI+MVI−), both microscopic bile duct and vascular invasion (MiBDI+MVI+), microscopic vascular invasion alone (MiBDI−MVI+), and macroscopic bile duct invasion (MaBDI). Overall survival (OS) was assessed using Kaplan–Meier analysis, and independent risk factors of OS were determined using Cox proportional hazards models.

**Results:**

A total of 377 HCC cases were analyzed. The OS for MiBDI+MVI‐ was similar to that of MiBDI−MVI− (*p* > 0.05) but better than MiBDI+MVI+, MiBDI−MVI+, and MaBDI (all *p* < 0.05). Multivariate analysis indicated that MiBDI was not an independent risk factor for OS, while MVI and MaBDI were.

**Conclusions:**

Overall survival (OS) in patients with MiBDI was superior to those with MVI and MaBDI. Isolated MiBDI did not influence OS in patients with HCC after R0 resection.

## 1 INTRODUCTION

Hepatocellular carcinoma (HCC) is the predominant form of primary liver cancer, ranking fourth in morbidity and second in mortality among all cancers.^1^ Hepatocellular carcinoma (HCC) is characterized by significant malignancy and strong invasiveness, making it prone to invasion and metastasis during tumor initiation and progression. Tumor cells infiltrate blood vessels, leading to vascular invasion or vascular tumor thrombus. While tumor cells infiltrate bile duct, leading to bile duct invasion (BDI) or bile duct tumor thrombus (BDTT).

Among these distinct anatomical invasion types, clinical research on vascular invasion is more comprehensive. Histopathological classifications of vascular invasion typically differentiate between microscopic (MVI) and macroscopic tumor thrombi (MaVI). MVI and MaVI have reported incidence rates of 15%–75% and 44%–62.2%, respectively.^2^, ^3^ Both two types of vascular invasion are considered independent prognostic factors for poor outcomes in HCC and are incorporated into the 8th edition of the TNM staging system as essential elements for predicting prognosis and informing treatment decisions.^4^ In the 8th edition TNM staging, patients with either MVI or MaVI are classified as stage II and stage IIIB, respectively. Conversely, the incidence of BDI is lower, with notably fewer studies compared with vascular invasion. To date, researches on BDI primarily originated from small sample‐size or case report‐based retrospective studies.

As research has advanced, clinicians and pathologists have enhanced their understanding of BDI. In recent years, the detection rate of BDI has been increasing.^5^ According to the literature, the clinical incidence of BDI ranges from 0.45% to 12.9%.^5^, ^6^, ^7^, ^8^, ^9^ Some retrospective studies have confirmed that patients with BDI experience shorter survival times and higher recurrence rates compared to those without BDI.^8^, ^10^, ^11^, ^12^, ^13^, ^14^ For patients with BDI who receive only palliative care or the best supportive treatment, the median survival interval is a mere 1.6–4.3 months.^15^, ^16^ Consequently, BDI is considered a risk factor affecting HCC prognosis, and several studies have confirmed that its prognosis is better than that of MaVI.^17^, ^18^ However, unlike MVI, only a few single‐center, small sample‐size retrospective studies exist on microscopic bile duct invasion (MiBDI). A retrospective study from Japan suggested that, compared with MiBDI, macroscopic bile duct invasion (MaBDI) was a protective factor for prognosis.^19^ A matched study from South Korea indicated that, compared with HCC without BDI, the prognosis of MiBDI was worse.^20^ A retrospective propensity score matching study from China revealed that the prognosis of MaBDI was worse than that of MiBDI, and the overall survival did not significantly differ between HCC without BDI and HCC with MiBDI.^21^ The prognostic differences MiBDI and MaBDI remain a subject of debate. As for distinctions between MiBDI and MVI, no relevant research has been conducted to date.

The aim of the present study is to distinguish the survival differences of patients with MiBDI, MaBDI, and MVI, and to evaluate the prognosis significance of MiBDI for HCC.

## 2 PATIENTS AND METHODS

### 2.1 Ethics statement

This study was conducted in accordance with the Declaration of Helsinki and the Ethical Guidelines for Clinical Research. Informed consent was obtained from all patients before surgery. Approval was obtained from the Institutional Research Ethics Committee of Mengchao Hepatobiliary Hospital of Fujian Medical University, with the approval number 2020_077_01. The data are anonymous, and the requirement for informed consent was therefore waived.

### 2.2 Patients

This retrospective observational study enrolled HCC patients with microscopic or macroscopic bile duct invasion (BDI) from nine Chinese hepatobiliary medical centers (Mengchao Hepatobiliary Hospital of Fujian Medical University, First Affiliated Hospital of Fujian Medical University, First Affiliated Hospital of Xiamen University, Affiliated Hospital of Qingdao University, Southwest Hospital of Army Medical University, Zhangzhou Affiliated Hospital of Fujian Medical University, Huashan Hospital of Fudan University, Sir RunRun Shaw Hospital of Zhejiang University School of Medicine, and Eastern Hepatobiliary Surgery Hospital of Second Military Medical University). The study was conducted between January 1, 2008, and December 31, 2019. Additionally, patients with HCC without BDI and HCC with MiVI from Mengchao Hepatobiliary Hospital of Fujian Medical University between January 1, 2015, and December 31, 2015, were also enrolled.

The inclusion criteria for this study were as follows: (1) confirmation of HCC and BDI by histopathology, (2) receipt of R0 resection, and (3) availability of complete clinical data and postoperative follow‐up records. The exclusion criteria were as follows: (1) combined HCC intrahepatic cholangiocarcinoma, (2) recurrent or metastatic HCC, (3) presence of portal vein tumor thrombus (PVTT), or (4) co‐occurrence with other malignancies. R0 resection was defined as the microscopically negative margin. Patients were further categorized based on their bile duct and vascular invasion status: no bile duct or vascular invasion (MiBDI−MVI−), microscopic bile duct invasion only (MiBDI+MVI−), both microscopic bile duct and vascular invasion (MiBDI+MVI+), microscopic vascular invasion only (MiBDI−MVI+), and macroscopic bile duct invasion (MaBDI).

### 2.3 Clinicopathological characteristics

The clinicopathological characteristics that were included in the study were age, gender, presence of liver cirrhosis, history of hepatitis B virus infection, number of tumors, tumor size, presence of satellite nodules, tumor differentiation grade, and microvascular invasion (MVI). Cirrhosis was confirmed either by histopathology or by clinical diagnosis. Tumor differentiation grade was classified according to the Edmonson–Steiner grade. MVI was defined as vascular invasion of small vessels discovered under the microscope. Referring to the definition of MVI in the clinical guidelines,^22^ the MiBDI was defined as the mass of cancer cells observed in the bile duct cavity under the microscope.

### 2.4 Treatments

The therapeutic schedule for patients in this study was determined by a multidisciplinary team at each hepatobiliary medical center. The decision to hepatectomy was based on the clinical tumor stage, liver function, and residual liver volume of the patients.

### 2.5 Postoperative Follow‐up

All patients underwent REGULAR follow‐up appointments after discharge, scheduled at 3‐month intervals for the first 2 years, at 6‐month intervals for 2–5 years, and annually thereafter. Follow‐up examinations included laboratory tests for tumor biomarkers, coagulation function, liver function, and whole blood count, as well as abdominal ultrasound and/or contrast‐enhanced computed tomography or magnetic resonance imaging. Overall survival (OS) was defined as the time from surgical resection to death or last follow‐up, and follow‐up for this study was censored on March 31, 2022.

### 2.6 Statistical analysis

Continuous variables were presented as the median with quartile ranges and were compared using either Student's *t*‐test or Mann–Whitney test. Categorical data were expressed as frequency (%) and were compared using either the chi‐squared test or Fisher's exact test. Overall survival (OS) was evaluated using Kaplan–Meier survival curves. Univariate and multivariable analyses were performed using the Cox proportional hazard regression model, with the backward stepwise selection method used to determine independent prognostic factors. Statistical significance was defined as *p*‐values less than 0.05. The statistical analysis was conducted using R version 4.2.2 (http://www.r‐project.org/) and IBM SPSS Statistics software (version 24.0; IBM Corp., Armonk, NY). The R packages utilized were “readxl,” “readr,” “table 1,” “ggpubr,” “plyr,” “survminer,” “survival,” and “ggplot2.

## 3 RESULTS

### 3.1 Patients' characteristics

The study enrolled 377 cases of HCC who received R0 resection, including 139 cases in the group with neither bile duct nor vascular invasion (MiBDI−MVI−), 11 cases in the group with microscopic bile duct invasion alone (MiBDI+MVI−), 37 cases in the group with both microscopic bile duct and microscopic vascular invasion (MiBDI+MVI+), 56 cases in the group with microscopic vascular invasion alone (MiBDI−MVI+), and 156 cases in the group with macroscopic bile duct invasion (MaBDI). The patients' characteristics are detailed in Table 1.

**TABLE 1:** Patients’ characteristics.

Characteristics	MiBDI−MVI−	MiBDI+MVI−	MiBDI+MVI+	MiBDI−MVI+	MaBDI
	(*N* = 139)	(*N* = 11)	(*N* = 37)	(*N* = 56)	(*N* = 134)
Age					
Median [IQR]	51.0 [43.0, 59.0]	52.0 [46.5, 57.0]	57.0 [52.0, 64.0]	50.5 [44.8, 59.0]	52.5 [46.3, 58.8]
Gender					
Female	12 (8.6%)	4 (36.4%)	8 (21.6%)	6 (10.7%)	19 (14.2%)
Male	127 (91.4%)	7 (63.6%)	29 (78.4%)	50 (89.3%)	115 (85.8%)
HBV					
No	32 (23.0%)	2 (18.2%)	6 (16.2%)	8 (14.3%)	30 (22.4%)
Yes	107 (77.0%)	9 (81.8%)	31 (83.8%)	48 (85.7%)	104 (77.6%)
Cirrhosis					
No	55 (39.6%)	4 (36.4%)	7 (18.9%)	19 (33.9%)	2 (1.5%)
Yes	84 (60.4%)	7 (63.6%)	30 (81.1%)	37 (66.1%)	132 (98.5%)
Child–Pugh grade					
A	136 (97.8%)	11 (100%)	30 (81.1%)	50 (89.3%)	95 (70.9%)
B	3 (2.2%)	0 (0%)	7 (18.9%)	6 (10.7%)	39 (29.1%)
TBil					
Median [IQR]	13.4 [11.3, 17.0]	13.5 [11.0, 33.3]	17.3 [11.9, 27.2]	12.2 [9.40, 15.9]	33.3 [17.6, 79.2]
AFP					
Median [IQR]	10.0 [4.20, 95.8]	121 [55.4, 782]	88.3 [12.0, 697]	34.8 [8.10, 1210]	15.7 [4.45, 423]
Tumor number					
Single	117 (84.2%)	11 (100%)	30 (81.1%)	39 (69.6%)	117 (87.3%)
Multiple	22 (15.8%)	0 (0%)	7 (18.9%)	17 (30.4%)	17 (12.7%)
Tumor size					
≤5 cm	84 (60.4%)	8 (72.7%)	20 (54.1%)	28 (50.0%)	73 (54.5%)
>5 cm	55 (39.6%)	3 (27.3%)	17 (45.9%)	28 (50.0%)	61 (45.5%)
ES grade					
I/II	22 (15.8%)	0 (0%)	8 (21.6%)	0 (0%)	28 (20.9%)
III/IV	117 (84.2%)	11 (100%)	29 (78.4%)	56 (100%)	106 (79.1%)
Capsule					
No	122 (87.8%)	5 (45.5%)	15 (40.5%)	49 (87.5%)	108 (80.6%)
Yes	17 (12.2%)	6 (54.5%)	22 (59.5%)	7 (12.5%)	26 (19.4%)
Satellite					
No	130 (93.5%)	6 (54.5%)	21 (56.8%)	38 (67.9%)	93 (69.4%)
Yes	9 (6.5%)	5 (45.5%)	16 (43.2%)	18 (32.1%)	41 (30.6%)
MVI					
No	139 (100%)	11 (100%)	0 (0%)	0 (0%)	41 (30.6%)
Yes	0 (0%)	0 (0%)	37 (100%)	56 (100%)	93 (69.4%)

Abbreviations: AFP, alpha‐fetoprotein; ES grade, Edmondson‐Steiner grade; HBV, hepatitis B virus; IQR, interquartile range; MVI, microvascular invasion; TBil, total bilirubin.

### 3.2 Microscopic histopathological characteristics of the BDI and MVI

**FIGURE 1:**
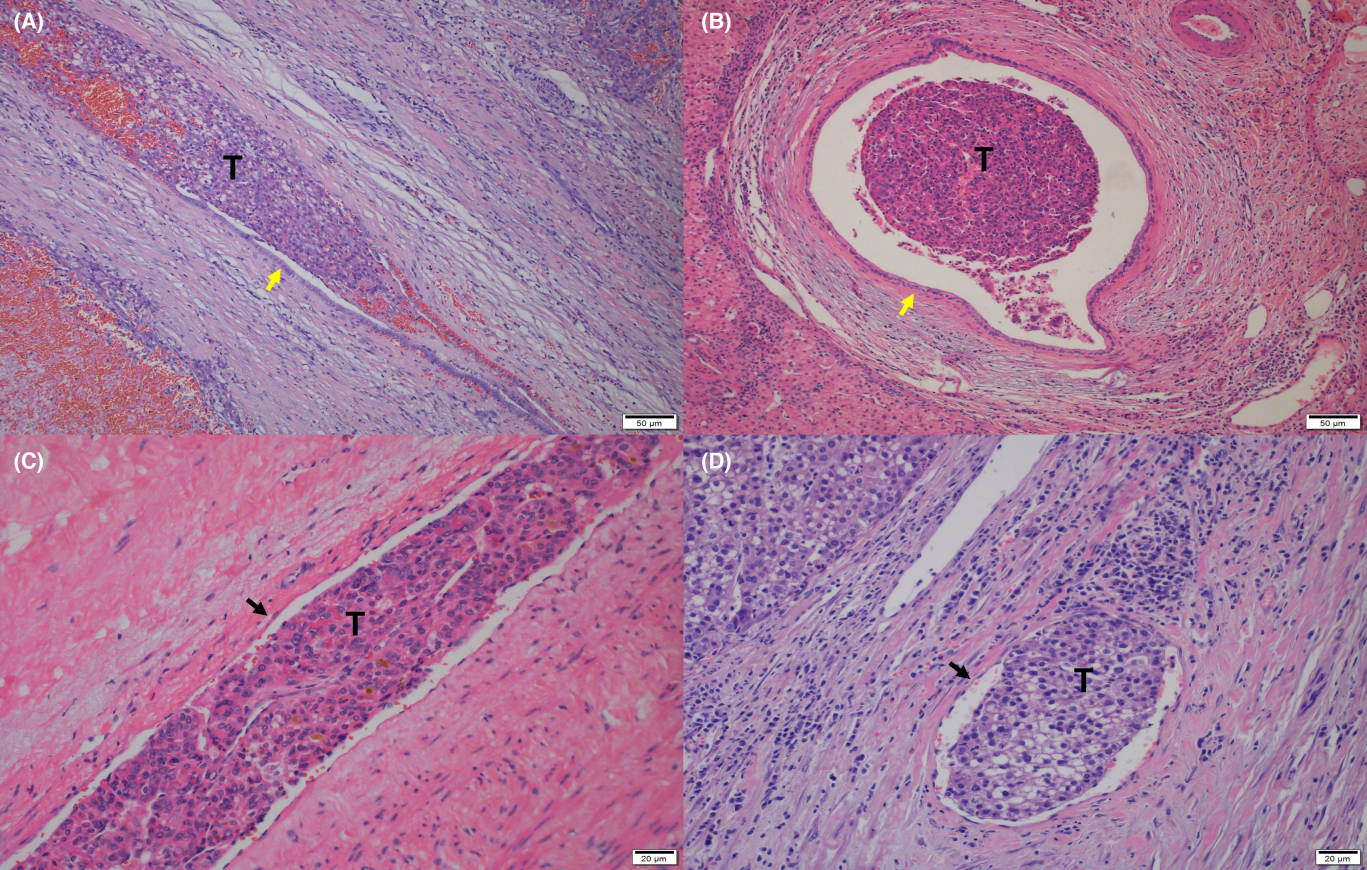
Microscopic histopathological characteristics of bile duct and microvascular; (A) longitudinal section of MiBDI (hematoxylin–eosin staining, ×100), (B) transection of MiBDI (hematoxylin–eosin staining, ×100), (C) longitudinal section of MVI (hematoxylin–eosin staining, ×200), and (D) transection of MVI (hematoxylin–eosin staining, ×200). Yellow arrow marked bile duct epithelium, black arrow marked vascular epithelium, and “T” means tumor.

Significant differences were observed between the microscopic features of bile duct and microvascular. The interlobular bile duct wall comprises epithelium and fibrous membrane, with the epithelium gradually transitioning from a single cuboidal to a single columnar shape, and an oval nucleus located at the base of the cell (Figure 1A,B). In contrast, the vascular intima consists of endothelium and subendothelium, with the endothelium lining the vascular lumen as a single layer of flat epithelium. The nucleus is located in the middle, lightly stained, and the edge is thin (Figure 1C,D).

### 3.3 Overall survival

**FIGURE 2:**
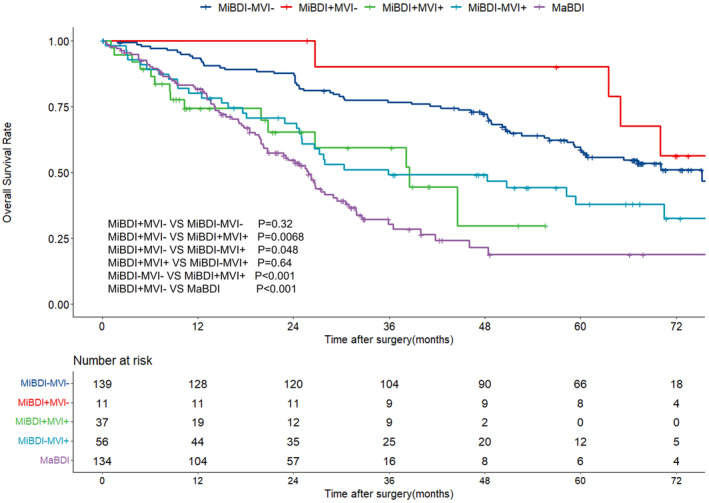
Kaplan–Meier survival curves of HCC with different tumor invasion status.

The median follow‐up time for the entire cohort was 61.50 months (95% confidence interval [CI], 57.28–67.72 months). The median OS was 75.23 months (95% CI 63.13–87.33 months) for the MiBDI−MVI− group, 85.00 months (95% CI 62.49–107.51 months) for the MiBDI+MVI− group, 38.53 months (95% CI, 20.22–56.84 months) for the MiBDI+MVI+ group, and 35.90 months (10.96–60.84 months) for the MiBDI−MVI+ group, respectively. The 1‐, 3‐, and 5‐year OS rates were 93.4%, 76.6%, and 58.6% for the MiBDI−MVI− group, 100%, 90%, and 90% for the MiBDI+MVI− group, 74.3%, 59.3%, and unreached for the MiBDI+MVI+ group, 80%, 49.1%, and 37.8% for the MiBDI−MVI+ group, and 81.7%, 30.2%, and 18.8% for the MaBDI group, respectively. The differences between groups were as follows: MiBDI+MVI− vs. MiBDI−MVI− (*p* = 0.32), MiBDI+MVI− vs. MiBDI+MVI+ (*p* = 0.0068), MiBDI+MVI− vs. MiBDI−MVI+ (*p* = 0.048), MiBDI+MVI+ vs. MiBDI−MVI+ (*p* = 0.64), MiBDI−MVI− vs. MiBDI+MVI+ (*p* < 0.001), and MiBDI+MVI− vs. MaBDI (*p* < 0.001), respectively (see Figure 2).

### 3.4 Risk factors of overall survival

Univariate analysis revealed that the risk factors for OS included cirrhosis, AFP, total bilirubin, Child–Pugh score, tumor number, tumor size, satellite nodules, and tumor invasion status (all *p* < 0.05). Multivariate analysis adjusting for relevant covariates identified Child–Pugh score, tumor number, tumor size, and tumor invasion status as independent risk factors for OS (all *p* < 0.05). Notably, there was no significant difference in OS between the MiBDI−MVI− and MiBDI+MVI− groups (*p* = 0.868), indicating that isolated MiBDI has no impact on the OS of HCC. (Table 2). In addition, further COX multivariate analysis which included MiBDI (*n* = 48), MVI (*n* = 186), and MaBDI (*n* = 134) as variables was conducted and the result still showed that MiBDI was not but MVI and MaBDI were the risk factors of OS (Table S1).

**TABLE 2:** Univariate and multivariate analysis of overall survival.

Characteristics	Univariate			Multivariate		
	HR	CI 95%	*p*‐value	HR	CI 95%	*p*‐value
Gender, male	1.183	0.771–1.814	0.441			
Age, per year	1.012	0.999–1.026	0.072	1.01	0.997–1.024	0.133
HBV, yes	1.198	0.841–1.708	0.317			
Cirrhosis, yes	1.879	1.311–2.693	0.001			
AFP, per	1	1.000–1.000	0.023			
TBIL, per	1.003	1.001–1.004	<0.001			
Child–Pugh, B	2.373	1.648–3.418	<0.001	1.547	1.044–2.291	0.03
Tumor number, multiple	1.847	1.299–2.625	0.001	1.791	1.252–2.561	0.001
Tumor size, >5 cm	2.084	1.567–2.771	<0.001	2.019	1.514–2.693	<0.001
Satellite	2.303	1.677–3.163	<0.001			
ES grade	1.195	0.795–1.794	0.392			
Capsule	0.785	0.542–1.137	0.2	0.701	0.473–1.04	0.077
Tumor invasion status	Reference (MiBDI−MVI−)			Reference (MiBDI−MVI−)		
MiBDI+MVI−	0.631	0.229–1.737	0.372	0.916	0.326–2.571	0.868
MiBDI+MVI+	2.597	1.458–4.628	0.001	2.603	1.397–4.848	0.003
MiBDI−MVI+	1.852	1.207–2.842	0.005	1.672	1.081–2.587	0.021
MaBDI	3.252	2.291–4.615	<0.001	2.875	1.97–4.195	<0.001

Abbreviations: AFP, alpha‐fetoprotein; ES grade, Edmondson‐Steiner grade; HBV, hepatitis B virus; IQR, interquartile range; MVI, microvascular invasion; TBil, total bilirubin.

## 4 DISCUSSION

Bile duct invasion is a seldom encountered occurrence in clinical practice, with reported incidences ranging from 0.45% to 12.9% in the literature.^5^, ^6^, ^7^, ^8^, ^9^ The occurrence of microscopic bile duct invasion is even more infrequent. As a result, there has been limited focus on its diagnosis and treatment, leading to a scarcity of relevant studies. Clinicians possess inadequate understanding of this phenomenon and may mistakenly classify microscopic bile duct invasion as MVI.^23^ The misclassification of these two types of invasions is likely to result in an inaccurate prognostic evaluation and subsequently improper medical intervention. To our knowledge, there are no studies on the difference in prognosis between MVI and MiBDI. Given that the prognosis of patients with HCC relies on precise prognostic assessment and suitable treatment recommendations, we undertook this study to evaluate the prognostic significance of microscopic bile duct invasion.

A retrospective study showed that the prognosis of HCC with MaBDI was better than HCC with MiBDI,^19^ while a propensity score matching study came to the opposite conclusion.^21^ Our results show that microscopic bile duct invasion is distinct from MVI, both morphologically and prognostically. From a morphological standpoint, there are clear differences between MiBDI and MVI. The bile duct epithelium primarily consists of cuboidal and columnar epithelial cells, while the vascular endothelium is mainly composed of single‐layered, flat epithelial cells. These differences make it relatively easy to distinguish the two morphologically. Additionally, immunohistochemical staining can be used to differentiate between them. For instance, CD34 is associated with brownish‐yellow staining of the cell membrane, indicating the presence of vascular endothelial cells. Conversely, CK19 does not stain liver cells but can specifically stain bile duct epithelium.^24^ In the survival analysis, we aimed to exclude the influence of MVI by grouping the subjects based on different types of tumor invasion. Our findings revealed that the OS of MiBDI+MVI− was not significantly different from that of MiBDI−MVI− (*p* = 0.32), but it was better than the outcomes for MiBDI+MVI+ (*p* = 0.0068), MiBDI−MVI+ (*p* = 0.048), and MaBDI (*p* < 0.001). Further multivariate analysis confirmed these results. Consequently, we believe that pure microscopic bile duct invasion does not impact the overall survival of HCC patients after R0 resection.

Numerous studies have been conducted on vascular invasion. Research findings indicate that the prognosis of MVI is better than that of macrovascular invasion,^4^, ^22^ and the prognosis of MaBDI is more favorable than that of macrovascular invasion.^5^, ^25^, ^26^, ^27^ In this study, the prognosis of MiBDI is also superior to that of MVI and better than that of MaBDI. Therefore, we summarize this phenomenon as “microscopic invasion is better than macroscopic invasion, and bile duct invasion is better than vascular invasion.” As for the possible reasons why BDI has a more favorable prognosis than vascular invasion, we speculated that this is because the corresponding distal organs are different. The invasion of tumor cells into the bile ducts and subsequent entry into the intestine via bile, coupled with the unfavorable conditions for tumor growth provided by the digestive juices in the intestine and the tumor cells are excreted along with the feces, bile duct invasion is less prone to distant metastases. Conversely, tumor cells that infiltrate blood vessels could disseminate throughout the body via the bloodstream, leading to the formation of metastatic cancer in various organs such as the lungs, bones, and brain, consequently impairing relevant organs.

This study has the following limitations: First, in the real world, there are far more HCC patients without BDI than those with BDI. To increase the sample size, we collected data through a multicenter study. However, due to the substantial workload, it was not feasible to collect HCC cases without BDI from all centers within the same period as a control, leading to potential selection bias in this retrospective study. Second, the number of patients with pure microscopic bile duct invasion is relatively small (only 11 cases), necessitating a larger sample and a well‐designed clinical study for further validation. Lastly, the patients in this study are all from China, with most of them having hepatitis B infections. The prognostic role of microscopic bile duct tumor thrombus in non‐hepatitis B‐related liver cancer requires additional investigation.

## 5 CONCLUSION

The overall survival of patients with MiBDI was better than that of those with MVI and MaBDI. Isolated MiBDI invasion does not impact the overall survival of HCC patients after R0 resection.

## AUTHOR CONTRIBUTIONS

**Qizhen Huang:** Conceptualization (lead); data curation (equal); formal analysis (lead); investigation (lead); methodology (lead); project administration (lead); validation (equal); writing – original draft (lead); writing – review and editing (lead). **Kongying Lin:** Data curation (equal); formal analysis (equal); investigation (equal); methodology (lead); software (lead); writing – original draft (equal); writing – review and editing (equal). **Zhipeng Lin:** Data curation (equal); formal analysis (equal); software (equal); validation (equal); writing – original draft (equal). **Hongbin Ji:** Data curation (equal); formal analysis (equal); investigation (equal); project administration (equal); resources (equal); writing – review and editing (equal). **Xiaoyu Zhou:** Data curation (equal); formal analysis (equal); methodology (equal); software (equal); validation (equal); visualization (equal). **Bin Wang:** Data curation (equal); methodology (equal); writing – original draft (equal). **Yufeng Chen:** Data curation (equal); writing – review and editing (equal). **Chuandong Sun:** Data curation (equal); writing – review and editing (equal). **Shuguo Zheng:** Data curation (equal); writing – review and editing (equal). **Jinhong Chen:** Data curation (equal); writing – review and editing (equal). **Yifan Wang:** Data curation (equal); writing – review and editing (equal). **Yanming Zhou:** Data curation (equal); writing – review and editing (equal). **Weiping Zhou:** Data curation (equal); writing – review and editing (equal). **Yongyi Zeng:** Conceptualization (equal); project administration (lead); resources (equal); supervision (lead); writing – review and editing (lead).

## FUNDING INFORMATION

This study was supported by the Startup Fund for scientific research, Fujian Medical University (Grant number: 2021QH1158), the Science and Technology project of Fuzhou (Grant number: 2020‐S‐112), and the Key Clinical Specialty Discipline Construction Program of Fuzhou, Fujian, P.R.C (Grant number: 201912002).

## CONFLICT OF INTEREST STATEMENT

The research was conducted in the absence of any commercial or financial relationships that could be construed as a potential conflict of interest.

## Supporting information


Table S1.

## Data Availability

The datasets used in the present study are available from the corresponding author on reasonable request.

## References

[1] Bray F , Ferlay J , Soerjomataram I , Siegel RL , Torre LA , Jemal A . Global cancer statistics 2018: GLOBOCAN estimates of incidence and mortality worldwide for 36 cancers in 185 countries. CA Cancer J Clin. 2018;68(6):394‐424. 30207593 10.3322/caac.21492

[2] Kokudo T , Hasegawa K , Matsuyama Y , et al. Survival benefit of liver resection for hepatocellular carcinoma associated with portal vein invasion. J Hepatol. 2016;65(5):938‐943. 27266618 10.1016/j.jhep.2016.05.044

[3] Kokudo T , Hasegawa K , Yamamoto S , et al. Surgical treatment of hepatocellular carcinoma associated with hepatic vein tumor thrombosis. J Hepatol. 2014;61(3):583‐588. 24798618 10.1016/j.jhep.2014.04.032

[4] Amin MB , Edge SB , Greene FL , et al. AJCC Cancer Staging Manual. 8th ed. Springer Nature; 2017.

[5] Kim DS , Kim BW , Hatano E , et al. Surgical outcomes of hepatocellular carcinoma with bile duct tumor thrombus: a Korea‐Japan multicenter study. Ann Surg. 2020;271(5):913‐921. 30216223 10.1097/SLA.0000000000003014

[6] Chotirosniramit A , Liwattanakun A , Junrungsee S , Ko‐Iam W , Sandhu T , Lapisatepun W . The benefit of curative liver resection with a selective bile duct preserving approach for hepatocellular carcinoma with macroscopic bile duct tumor thrombus. Hepatobiliary Surg Nutr. 2020;9(6):729‐738. 33299828 10.21037/hbsn.2019.10.26 PMC7720058

[7] Satoh S , Ikai I , Honda G , et al. Clinicopathologic evaluation of hepatocellular carcinoma with bile duct thrombi. Surgery. 2000;128(5):779‐783. 11056440 10.1067/msy.2000.108659

[8] Shiomi M , Kamiya J , Nagino M , et al. Hepatocellular carcinoma with biliary tumor thrombi: aggressive operative approach after appropriate preoperative management. Surgery. 2001;129(6):692‐698. 11391367 10.1067/msy.2001.113889

[9] Zhang B , Zhang B , Zhang Z , et al. 42,573 cases of hepatectomy in China: a multicenter retrospective investigation. Sci China Life Sci. 2018;61(6):660‐670. 29417360 10.1007/s11427-017-9259-9

[10] Ikenaga N , Chijiiwa K , Otani K , Ohuchida J , Uchiyama S , Kondo K . Clinicopathologic characteristics of hepatocellular carcinoma with bile duct invasion. J Gastrointest Surg. 2009;13(3):492‐497. 19011945 10.1007/s11605-008-0751-0

[11] Meng KW , Dong M , Zhang WG , Huang QX . Clinical characteristics and surgical prognosis of hepatocellular carcinoma with bile duct invasion. Gastroenterol Res Pract. 2014;2014:604971. 24723944 10.1155/2014/604971 PMC3958710

[12] Pang YB , Zhong JH , Luo XL , et al. Clinicopathological characteristics and liver stem cell marker expression in hepatocellular carcinoma involving bile duct tumor thrombi. Tumour Biol. 2016;37(5):5879‐5884. 26586401 10.1007/s13277-015-4446-3

[13] Shao W , Sui C , Liu Z , Yang J , Zhou Y . Surgical outcome of hepatocellular carcinoma patients with biliary tumor thrombi. World J Surg Oncol. 2011;9:2. 21214943 10.1186/1477-7819-9-2 PMC3022747

[14] Wang DD , Wu LQ , Wang ZS . Prognosis of hepatocellular carcinoma with bile duct tumor thrombus after R0 resection: a matched study. Hepatobiliary Pancreat Dis Int. 2016;15(6):626‐632. 27919852 10.1016/s1499-3872(16)60143-1

[15] An J , Lee KS , Kim KM , et al. Clinical features and outcomes of patients with hepatocellular carcinoma complicated with bile duct invasion. Clin Mol Hepatol. 2017;23(2):160‐169. 28506055 10.3350/cmh.2016.0088 PMC5497660

[16] Peng BG , Liang LJ , Li SQ , Zhou F , Hua YP , Luo SM . Surgical treatment of hepatocellular carcinoma with bile duct tumor thrombi. World J Gastroenterol. 2005;11(25):3966‐3969. 15991304 10.3748/wjg.v11.i25.3966 PMC4504907

[17] Lu WP , Tang HW , Yang ZY , Jiang K , Chen YL , Lu SC . A proposed modification for the Barcelona clinic liver cancer staging system: adding bile duct tumor thrombus status in patients with hepatocellular carcinoma. Am J Surg. 2020;220(4):965‐971. 32336518 10.1016/j.amjsurg.2020.04.003

[18] Wu JY , Sun JX , Bai YN , et al. Long‐term outcomes of anatomic versus nonanatomic resection in hepatocellular carcinoma patients with bile duct tumor thrombus: a propensity score matching analysis. Ann Surg Oncol. 2021;28(12):7686‐7695. 33929619 10.1245/s10434-021-09874-3

[19] Esaki M , Shimada K , Sano T , Sakamoto Y , Kosuge T , Ojima H . Surgical results for hepatocellular carcinoma with bile duct invasion: a clinicopathologic comparison between macroscopic and microscopic tumor thrombus. J Surg Oncol. 2005;90(4):226‐232. 15906365 10.1002/jso.20260

[20] Kim JM , Kwon CHD , Joh JW , et al. Incidental microscopic bile duct tumor thrombi in hepatocellular carcinoma after curative hepatectomy: a matched study. Medicine (Baltimore). 2015;94(6):e450. 25674733 10.1097/MD.0000000000000450 PMC4602767

[21] Yang X , Qiu Z , Ran R , et al. Prognostic importance of bile duct invasion in surgical resection with curative intent for hepatocellular carcinoma using PSM analysis. Oncol Lett. 2018;16(3):3593‐3602. 30127966 10.3892/ol.2018.9108 PMC6096155

[22] Zhou J , Sun H , Wang Z , et al. Guidelines for the diagnosis and treatment of hepatocellular carcinoma (2019 edition). Liver Cancer. 2020;9(6):682‐720. 33442540 10.1159/000509424 PMC7768108

[23] Sun Z , Shao WW , Song J . Recent advance in diagnosis and treatments for hepatocellular carcinoma with microvascular invasion. Chin J Hepat Surg(Electronic Edition). 2021;10(3):235‐241.

[24] Yu XH , Xu LB , Liu C , Zhang R , Wang J . Clinicopathological characteristics of 20 cases of hepatocellular carcinoma with bile duct tumor thrombi. Dig Dis Sci. 2011;56(1):252‐259. 20437099 10.1007/s10620-010-1256-8

[25] Huang Q , Chen Y , Lin K , et al. Redefining hepatocellular carcinoma staging systems based on the bile duct invasion status: a multicenter study. Front Oncol. 2021;11:673285. 34722235 10.3389/fonc.2021.673285 PMC8551376

[26] Kasai Y , Hatano E , Seo S , Taura K , Yasuchika K , Uemoto S . Hepatocellular carcinoma with bile duct tumor thrombus: surgical outcomes and the prognostic impact of concomitant major vascular invasion. World J Surg. 2015;39(6):1485‐1493. 25651961 10.1007/s00268-015-2985-9

[27] Noda T , Nagano H , Tomimaru Y , et al. Prognosis of hepatocellular carcinoma with biliary tumor thrombi after liver surgery. Surgery. 2011;149(3):371‐377. 20869094 10.1016/j.surg.2010.08.006

