# Supplementary material for: Prognosis value of microscopic bile duct invasion in hepatocellular carcinoma: A multicenter study
Source: Cancer Med. 2023 Nov 1;12(22):20821–9. doi: 10.1002/cam4.6650 (PMC10709741; doi:10.1002/cam4.6650)
Supplement: Supplementary file 1 — Table S1. [file CAM4-12-20821-s001.docx]

| Supplementary Table 1 Univariate and multivariate analysis of overall survival | | | | | | | |
| --- | --- | --- | --- | --- | --- | --- | --- |
| Characteristics | Univariate | | |  | Multivariate | | |
|  | HR | CI95% | P-value |  | HR | CI95% | P-value |
| Gender,Male | 1.183 | 0.771-1.814 | 0.441 |  |  |  |  |
| Age,per year | 1.012 | 0.999-1.026 | 0.072 |  | 1.012 | 0.999-1.026 | 0.079 |
| HBV, Present | 1.198 | 0.841-1.708 | 0.317 |  |  |  |  |
| Cirrhosis, Present | 1.879 | 1.311-2.693 | 0.001 |  |  |  |  |
| AFP, per | 1.000 | 1.000-1.000 | 0.023 |  |  |  |  |
| TBIL, per | 1.003 | 1.001-1.004 | <0.001 |  |  |  |  |
| Child-Pugh, B | 2.373 | 1.648-3.418 | <0.001 |  | 1.406 | 0.94-2.102 | 0.098 |
| Tumor Number, multiple | 1.847 | 1.299-2.625 | 0.001 |  | 1.731 | 1.213-2.47 | 0.002 |
| Tumor Size, >5cm | 2.084 | 1.567-2.771 | <0.001 |  | 2.023 | 1.518-2.695 | <0.001 |
| Satellite, Present | 2.303 | 1.677-3.163 | <0.001 |  |  |  |  |
| ES grade | 1.195 | 0.795-1.794 | 0.392 |  |  |  |  |
| Capsule, Present | 0.785 | 0.542-1.137 | 0.2 |  |  |  |  |
| MVI, Present | 2.424 | 1.811-3.246 | <0.001 |  | 1.748 | 1.266-2.415 | 0.001 |
| MiBDI, Present | 0.913 | 0.568-1.468 | 0.708 |  |  |  |  |
| MaBDI, Present | 1.263 | 1.171-1.362 | <0.001 |  | 1.174 | 1.08-1.275 | <0.001 |

AFP, alpha-fetoprotein; ES Grade, Edmondson-Steiner grade; HBV, Hepatitis B Virus; IQR, interquartile range; MVI, microvascular invasion; TBil, total bilirubin.
